# Physiotherapist prediction of extubation outcome in the adult intensive care unit

**DOI:** 10.1002/pri.1793

**Published:** 2019-06-25

**Authors:** Gabriella Cork, Luigi Camporota, Leyla Osman, Harriet Shannon

**Affiliations:** ^1^ Department of Physiotherapy Guy's and St Thomas' NHS Foundation Trust London UK; ^2^ University College London Institute of Child Health UCL Great Ormond Street Institute of Child Health London UK; ^3^ School of Health Sciences University of Liverpool Liverpool UK; ^4^ Department of Adult Critical Care Guy's and St Thomas' NHS Foundation Trust London UK; ^5^ King's Health Partners, Division of Asthma, Allergy and Lung Biology King's College London London UK

**Keywords:** extubation, physiotherapy, respiratory therapy/methods (MeSH), ventilator weaning/ methods (MeSH)

## Abstract

**Objective:**

Most patients requiring intubation and mechanical ventilation are extubated successfully at the first attempt; however, a minority experience extubation failure, which is associated with increased risk of ventilator‐associated pneumonia, prolonged intensive care unit (ICU) length of stay and mortality. Physiotherapists have expertise to assess cough strength, work of breathing, respiratory muscle strength, and respiratory secretion load, which are important factors in the outcome of extubation. Accurate prediction of extubation outcome could help to inform management plans pre‐extubation and postextubation. The primary objective of this service evaluation was to report the accuracy of physiotherapists' prediction of extubation outcome in the adult ICU.

**Methods:**

A single‐centre case note review was undertaken. All subjects who received a physiotherapy assessment of extubation suitability prior to extubation between January and March 2016 in the adult ICU of a large teaching hospital in the United Kingdom were included. Assessment, by both specialist and nonspecialist physiotherapists—which included risk stratification of extubation failure as “high,” “moderate,” or “low”—was undertaken prior to extubation. Logistic regression analysis was performed to determine which pre‐extubation factors were predictive of extubation outcome.

**Results:**

During the evaluation period, 68 subjects were extubated following a physiotherapy assessment. Physiotherapy risk stratification as “high risk” (OR 4; 95% confidence interval, CI, [1.312]; p=0.009) and “inappropriate” neurological status (OR 3.3; 95% CI [1.0410]; p=0.037) were the only pre‐extubation factors significantly associated with extubation failure. Assessment by specialist physiotherapists demonstrated greater sensitivity (100% vs. 22%) but lower specificity (68% vs. 95%) to detect extubation failure compared with the assessment performed by nonspecialist physiotherapists.

**Conclusion:**

Patients classified as “high risk” of extubation failure by a physiotherapist are significantly more likely to fail extubation. Specialist physiotherapists should be involved in the decision to extubate patients in the adult ICU.

## 1 INTRODUCTION

Approximately 180,000 patients are admitted to an intensive care unit (ICU) in the United Kingdom per year, and up to 70% of these will require intubation and mechanical ventilation during their stay (Simpson, Ross, McKeown, & Ray, 2012). Most patients who require intubation are extubated successfully at the first attempt. Early extubation, where feasible, is important as prolonged intubation and mechanical ventilation are associated with increased incidence of ventilator‐associated pneumonia (VAP), airway trauma, increased ICU length of stay (LOS), and increased mortality (Menon et al., 2012; Thille, Richard, & Brochard, 2013; Zilberberg, Kramer, Higgins, & Shorr, 2009).

Conversely, if patients are extubated too early, independent ventilation may not be sustained with 15–30% of patients suffering prolonged or complex weaning that can include extubation failure, reintubation, and/or tracheostomy (Boles et al., 2007). Extubation failure is defined as the need for reintubation within an arbitrary timescale, commonly 48–72 hr postextubation (Thille, Harrois, Schortgen, Brun‐Buisson, & Brochard, 2011). Recent studies describing the use of non‐invasive ventilation (NIV) as a planned bridge to extubation or a rescue therapy following extubation have led to the inclusion of late extubation failure (up to 7 days postextubation) within the definition (Girault et al., 2011; Thille et al., 2016). Extubation failure is associated with increased incidence of VAP, increased ICU‐LOS, and up to 50% increased mortality (Frutos‐Vivar et al., 2011; Menon et al., 2012; Thille et al., 2011; Torres et al., 1995). Therefore, the assessment of the patient's ability to breathe without assistance, their readiness for extubation, and their risk of failure are of crucial importance in determining the earliest time for successful extubation.

Respiratory physiotherapy involves comprehensive assessment of the patient's respiratory function, including breathing pattern and respiratory muscle function (European Respiratory Society, 2013). Cough strength (Smailes, McVicar, & Martin, 2013), work of breathing (Vallverdu et al., 1998), respiratory muscle strength (Bruton, 2002), and respiratory secretion load (Khamiees, Raju, DeGirolamo, Amoateng‐Adjepong, & Manthous, 2001) have been suggested as important factors in the outcome of extubation. Consequently, physiotherapists are well placed to contribute to the assessment of the patient's suitability for extubation. Physiotherapists also have a role in supporting patients at high risk of reintubation following extubation, with techniques including augmented cough (Berney, Stockton, Berlowitz, & Denehy, 2002) and adjuncts such as NIV and mechanical in‐exsufflation (Bach, Goncalves, Hamdani, & Winck, 2010; Vianello et al., 2011), with previous research concluding that physiotherapy following extubation prevents reintubation (Hanekom, Louw, & Coetzee, 2012). Accurate assessment methods for classifying patients as high risk prior to extubation would ensure that physiotherapy resources are directed towards those that would benefit from targeted, problem‐based intensive postextubation support.

A number of models have been developed in an attempt to find a quantitative method for predicting extubation success (Nemer & Barbas, 2011); however, clinical acumen is an important feature of clinical practice. Thille et al. (2015) reported that the predictive accuracy of nursing and medical staff regarding extubation outcome following a bedside assessment is low (only 34% of patients who failed extubation had been considered at high or very high risk of extubation failure). A predictive model utilizing cough strength, secretion abundance, and mechanical ventilation duration outperformed the clinician's acumen; however, the opinions of physiotherapists regarding suitability for extubation were not sought. Physiotherapists have a unique skill set that combines specific assessment skills, advanced clinical reasoning, and targeted interventions related to secretion management during the patient's intubation phase. As a result, they are well placed to predict extubation outcome and may offer additional insights compared with those health professionals studied by Thille et al. (2015). Moreover, because physiotherapists are involved in the respiratory secretion management of patients both before and after extubation, they are in an ideal position to contribute to decision making regarding extubation readiness and risk.

The aim of this service evaluation was to determine whether, following an assessment of extubation suitability, physiotherapists could correctly predict the extubation outcome of intubated adults in the ICU. Secondary objectives included determining whether specialist and nonspecialist physiotherapists differed in their predictive accuracy and whether any individual items in the physiotherapy assessment were associated with extubation outcome.

## 2 METHOD

### 2.1 Design and setting

Single‐centre case note review undertaken as a service evaluation

The project qualified as a service evaluation as defined by the UK NHS Health Research Authority and therefore did not require review by the Research Ethics Committee (http://www.hra.nhs.uk). It received institutional approval (institutional governance reference number 5893) and the need for individual informed consent was waived.

St. Thomas' Hospital is a large U.K. teaching hospital, providing 30 adult ICU beds. The case mix is largely medical and emergency surgical. Physiotherapy provision consists of four specialist physiotherapists and six nonspecialist physiotherapists. Specialist physiotherapists have over 5 years of postqualification experience and work exclusively in adult ICU, whereas nonspecialists undertake 4‐ to 6‐monthly rotations in ICU as part of their postqualification training. These staff provide both respiratory and rehabilitative interventions for both intubated and spontaneously breathing patients across 7 days, 11.5 hr per day with on‐call respiratory physiotherapy available overnight.

### 2.2 Subjects

Admissions records were screened for all patients admitted to a level three adult ICU between January 1, 2016 and March 31, 2016. All subjects who were intubated during the evaluation period and received a physiotherapy assessment of extubation suitability prior to extubation were eligible for inclusion. Subjects were excluded if they were extubated without a physiotherapy assessment, extubated prior to ICU admission, tracheotomized without a trial of extubation, died prior to their extubation attempt, or their extubation was deemed to be palliative or one way by the ICU consultant. A convenience sample was obtained during the designated timescale for the service evaluation, with the target of reporting at least 100 extubation events. This was comparable with numbers obtained in studies regarding prediction of extubation outcome (Meade et al., 2001) and provided sufficient data for statistical analyses.

### 2.3 Procedure

#### 2.3.1 Physiotherapy assessment procedure

As per usual practice, intubated patients, whose presenting conditions had resolved and were for consideration of extubation, were identified at daily multidisciplinary handover meetings. Where possible, patients were assessed by a physiotherapist for extubation suitability, although they were extubated without this if the assessment would delay extubation. Physiotherapy assessment occurred during a sedation hold, whilst maintaining good gas exchange and with minimal support during continuous spontaneous ventilation Positive end expiratory pressure (PEEP) ≤ 8 cmH_2_O; pressure support ≤7 cmH_2_O; FiO_2_ ≤ 0.4; SaO_2_ > 90%; and PaO_2_ > 8 kPa). The median of three pressures at the airways during the first 100 ms of inspiration (P_0.1_) were recorded using the appropriate function of the ventilator (Vallverdu et al., 1998). Maximal inspiratory pressure (MIP; PI_max_) was measured using the designated function of the ventilator. A 20‐s expiratory hold was performed, and the most negative reading is recorded (Bruton, 2002). Rapid shallow breathing index (RSBI) was calculated automatically by the ventilator as the ratio between respiratory rate and tidal volume (Yang & Tobin, 1991). Respiratory secretion burden was assessed as “minimal, moderate or copious,” by sputum yield during suction or airway clearance techniques, if required (Khamiees et al., 2001). The patient's cough strength was assessed during a volitional or spontaneous cough and measured by recording the peak cough expiratory flow (PCEF) on the ventilator flow waveform (Su et al., 2010). Appropriate neurological status was assessed by the ability of the patient to follow simple commands (e.g., tracking with their eyes and squeezing their hands; Salam, Tilluckdharry, Amoateng‐Adjepong, & Manthous, 2004). Following physiotherapy assessment, an unsupported breathing trial was implemented without PEEP, pressure support, or automatic tube compensation for 30 min. Criteria for unsupported breathing trial failure are summarized in Table 1.

**Table 1:** Unsupported breathing trial failure criteria

The new onset of any one of the following:
Physiological assessment:
• Heart rate > 20% of baseline or >140 beats per min
• Systolic BP >20% of baseline or >180 mmHg or <90 mmHg
• Cardiac arrhythmias
• Respiratory rate > 50% of baseline value or >35 per min
• Respiratory rate (min)/tidal volume (L) >105 per min per litre
Arterial blood gases:
• PaO_2_ < 8 kPa on FiO_2_ > 0.5 or (SpO_2_ < 90%)
• PaCO_2_ > 6.5 kPa or increase by >1 kPa
• pH <7.32 or fall by >0.07 units
Clinical assessment:
• Agitation and anxiety
• Depressed mental status
• Sweating/clammy
• Cyanosis
• Increased respiratory effort (accessory muscles, facial distress, and dyspnoea)

Following assessment of the above parameters, and prior to extubation, the physiotherapist stratified the patient's risk of extubation failure using their own clinical acumen into “low, moderate or high” risk and discussed this with the ICU consultant who made the final decision of whether to extubate the patient. This reflects typical clinical practice of consultant‐led extubation in U.K. ICUs.

#### 2.3.2 Case note review procedure

Patients' electronic health records (CareVue Philips Medical Systems UK Limited) were scrutinized manually. For all subjects, specialism of physiotherapist (specialist or nonspecialist physiotherapist), sputum load, appropriateness of neurology, PCEF, P0.1, mean inspiratory pressure, outcome of unsupported breathing trial, and the physiotherapist's risk stratification were recorded prior to extubation as per usual practice. Age, presenting condition, pre‐existing chronic lung or cardiac disease (Thille et al., 2011), ICU‐LOS, duration of intubation, weaning classification (simple, difficult, or prolonged according to definitions by Boles et al., 2007), ICU survival, and severity score (Acute Physiology and Chronic Health Evaluation) were documented retrospectively.

Extubation success was defined as a patient's ability to spontaneously breathe without NIV, with independent maintenance of their upper airway patency (no requirement for mechanical in‐exsufflation, artificial airway, or nasopharyngeal suction) at 1‐week postextubation. Extubation failure was therefore defined as reintubation up to 1 week following extubation, or ongoing requirement for NIV or airway clearance adjuncts at 1 week following extubation (Thille et al., 2016).

#### 2.3.3 Statistical analysis

Statistical analysis was performed using SPSS software (IBM SPSS Statistics Version 22). Sensitivity, specificity, negative and positive predictive values, and accuracy of the physiotherapists' high risk stratification to detect extubation failure were analysed. Quantitative variables for those who failed extubation were compared with those who were successful using two‐tailed Mann Whitney *U* tests or Student *t* tests for nonparametric or parametric data respectively, and chi squared for categorical data with significance value of <.05 accepted. Extubation failure rates for the three risk categories were compared using one‐way analysis of variance and post hoc Bonferroni's analysis.

Three logistic regression analyses were performed. First, univariate analysis of items from the physiotherapy assessment that were potentially associated with extubation outcome. Second, a multiple block regression analysis to determine whether a logistic regression predictor model could outperform the physiotherapist risk stratification. Third, binary categorization of physiotherapist risk stratification (high or moderate/low) as the dependent variable in univariate logistic regression investigated factors that weighted the physiotherapists' prediction of extubation outcome.

## 3 RESULTS

### 3.1 Subjects

**Figure 1:**
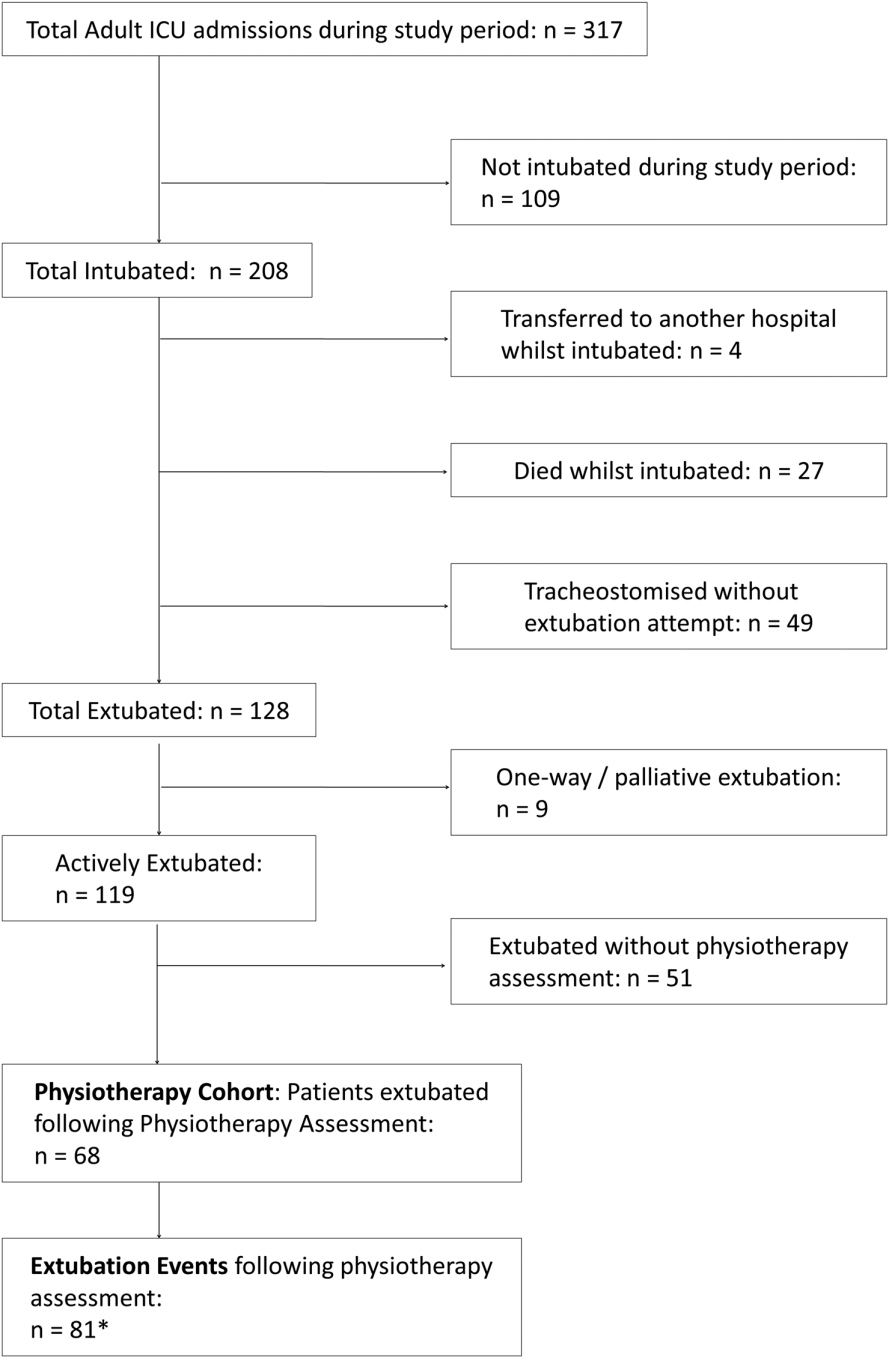
Service evaluation cohort identification* 12 subjects experienced multiple extubation events. ICU, intensive care unit

During the 3‐month evaluation period, 208 subjects were intubated and received mechanical ventilation. After exclusion criteria were applied, 68 subjects were actively extubated following a physiotherapy assessment of extubation suitability (Figure 1). Twelve subjects (18%) had repeated extubations (median 1 extubation per subject; interquartile range 1–2); therefore, in total, 81 extubation events were included. Nine different physiotherapists performed the assessments, four of whom were specialist intensive care physiotherapists.

Subjects (*n* = 68) were predominantly male (65%), with either medical conditions (68%) or following emergency surgery (32%), and there was a 43% prevalence of premorbid chronic cardiorespiratory disease (Table 2). Simple, difficult, and prolonged weaning accounted for 39(57%), 13(19%), and 16(26%) of the subjects, respectively. Extubation failure occurred in 20(29%) subjects, 14 of whom failed within 48 hr of extubation. Subjects who failed extubation had a significantly longer ICU‐LOS (17 days vs. 8 days; *p* = .002) and greater ICU mortality (7% vs. 0%; RR 24; 95% confidence interval, CI, [1.4, 420]; *p* = .001).

**Table 2:** Subject demographics

Variable	Subjects (*n* = 68)
Age (years)	58 ± 18
Gender	
Male	44 (65%)
Female	24 (35%)
Presenting condition	
Respiratory	19 (28%)
Pneumonia	14 (21%)
Neurology	5 (7%)
Other medical	8 (12%)
Emergency surgical	22 (32%)
Chronic cardiorespiratory disease	29 (43%)
APACHE II	17 ± 5
ICU‐LOS (days)	10 (6–16)
Intubation duration (days)	5 (4–8)
ICU mortality	5 (7%)
Extubation outcome:	
Extubation failure	20 (29%)
Extubation success	48 (71%)
Early Extubation Failure (≤48 hr)	14 (21%)
Late Extubation Failure (>48 hr)	6 (9%)
Weaning type:	
Simple	39 (57%)
Difficult	13 (19%)
Prolonged	16 (26%)

*Note*. Values are displayed as number (%), mean (±SD), or median (IQR).

Abbreviations: APACHE II, Acute Physiology and Chronic Health Evaluation version II; ICU‐LOS, intensive care unit length of stay; IQR, interquartile range.

### 3.2 Prediction of extubation outcome

**Figure 2:**
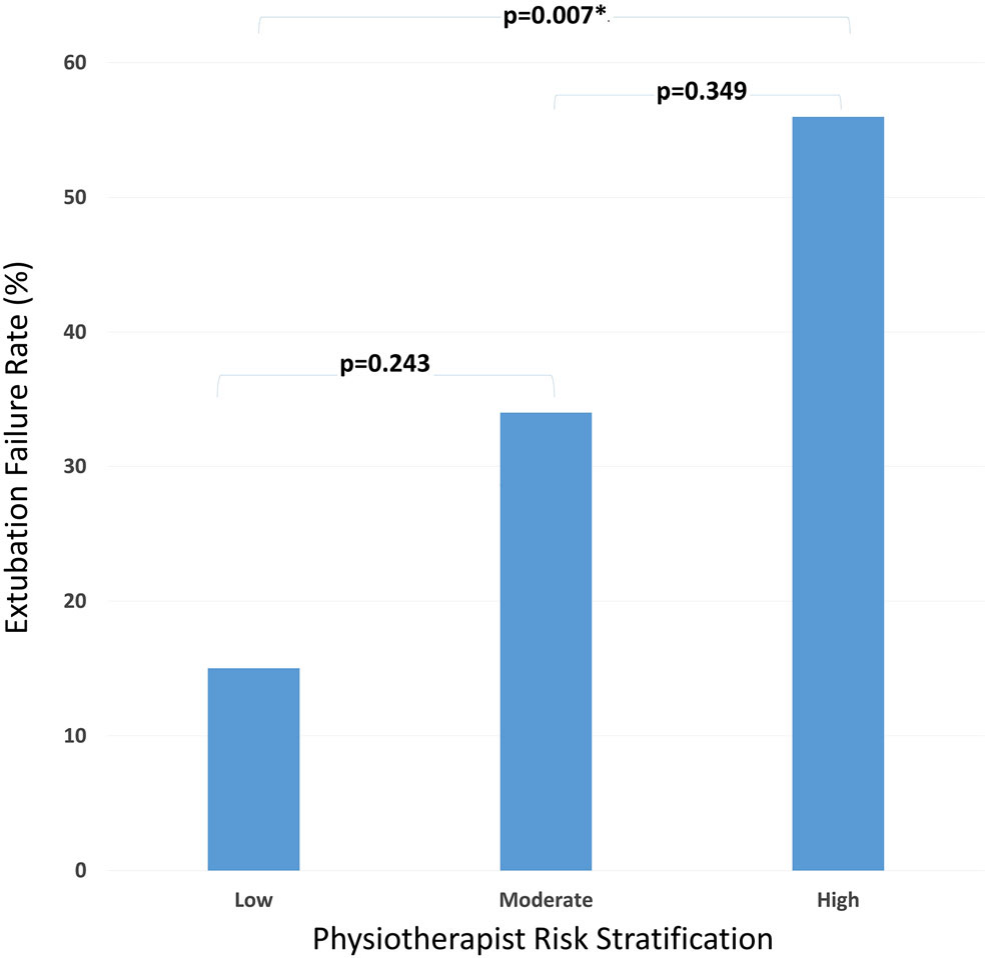
Extubation failure rate per physiotherapist risk stratification category

Extubation failure rates for extubations predicted as low, moderate, and high risk were 15%, 34%, and 56%, respectively (one‐way analysis of variance, *p* = .007; Figure 2). Individual items in the physiotherapy assessment prior to each extubation event are compared for extubation success and failure in Table 3.

**Table 3:** Physiotherapy assessment items for extubation failure compared with extubation success

Variable	Total (*n* = 81)	Extubation success (*n* = 56)	Extubation failure (*n* = 25)	*p* value	Mean/median difference or odds ratio	95% CI
Inappropriate neurology	15 (19%)	7 (13%)	8 (32%)	*p* = .037*	3.3	[1.04, 10]
Abundance of secretions						
Minimal	42 (51%)	31 (55%)	11 (44%)		0.63	[0.24, 1.6]
Moderate	32 (40%)	21 (37%)	11 (44%)	*p* = .581	1.3	[0.5, 3.4]
Copious	7 (9%)	4 (7%)	3 (12%)		1.8	[0.4, 8.5]
PCEF (L min^−1^)	97 ± 34	99 ± 32	92 ± 38	*p* = .417	−6.65	[−22.9, 9.58]
P0.1 (cmH_2_O)	3.3 (2.1–4.75)	3.1 (2–4.6)	4.0 (2.2–5.5)	*p* = .172	0.7	[−0.3, 1.7]
MIP (cmH_2_O)	29 ± 11	30 ± 11	28 ± 10	*p* = .452	−2	[−3, 7]
RSBI (f/V_T_)	40 (27.5–55)	40 (25–58)	42 (32–55)	*p* = .602	3	[−7, 12]
Physiotherapy risk assessment						
Low risk	34 (42%)	29 (52%)	5 (20%)		0.23	[0.07, 0.7]
Moderate risk	29 (36%)	19 (34%)	10 (40%)		1.29	[0.49, 3.4]
High risk	18 (22%)	8 (14%)	10 (40%)	*p* = .009*	4	[1.3, 12]
Failed SBT	8 (10%)	5 (9%)	3 (12%)	*p* = .669	1.4	[0.3, 6.3]
Type of physiotherapist						
Specialized	25 (31%)	19 (34%)	6 (24%)		0.615	[0.21, 1.8]
Nonspecialized	56 (69%)	37 (66%)	19 (76%)	*p* = .372	1.63	[0.56, 4.7]

*Note*. Values are displayed as number (%), mean (±SD), or median (IQR). Odds ratios are calculated for proportions as odds of having this characteristic with extubation failure compared with extubation success.

Abbreviations: MIP, maximal inspiratory pressure; PCEF, peak cough expiratory flow; P0.1, occlusion pressure; RSBI, rapid shallow breathing index; SBT, spontaneous breathing trial.

* Statistical significance *p* < .05.

The predictive accuracy of physiotherapists' detection of extubation failure is presented in Table 4. Specialist physiotherapists yielded the highest sensitivity to detect extubation failure (fewest false negatives) as well as the highest overall accuracy (total correct predictions). A predictive model based on multiple logistic block regression of nine independent variables (age, presence of chronic cardiorespiratory disease, intubation duration, previous extubation failure, appropriate neurology, PCEF, MIP, RSBI, and spontaneous breathing trial failure) detected extubation failure with the highest specificity (fewest false positives).

**Table 4:** Prediction of extubation failure

Predictor	Sensitivity	Specificity	PPV	NPV	Accuracy
All PTs	40% (24–54)	86% (79–92)	56% (34–75)	76% (70–82)	72% (62–80)
Specialized PTs	100% (57–100)	68% (55–68)	50% (28–50)	100% (80–100)	76% (55–76)
Nonspecialized PTs	22% (8–31)	95% (88–89)	67% (25–94)	72% (67–75)	71% (62–77)
Logistic regression model	28% (15–34)	96% (91–99)	78% (42–96)	75% (70–77)	75% (67–79)

*Note*. Values are displayed as percentage (95% CI).

Abbreviations: NPV, negative predictive value; PPV, positive predictive value; PT, physiotherapist.

Following univariate logistic regression analysis of the same nine potentially predictive variables, two were significantly associated with extubation outcome. Subjects who were classified by physiotherapists as “high risk” were four times more likely to fail an attempt at extubation compared with subjects classified as moderate/low risk (OR 4; 95% CI [1.34, 12]). Similarly, subjects with an “inappropriate” neurological status were three times more likely to fail extubation compared with appropriate neurology (OR 3.3; 95% CI [1.04, 10]).

Variables that were significantly associated with physiotherapist high risk classification were duration of intubation (in days; OR 1.16; 95% CI [1.05, 1.29]), copious secretions (OR 12; 95% CI [2, 67]), RSBI (OR 1.03; 95% CI [1.01, 1.06]), and spontaneous breathing trial failure (OR 39; 95% CI [4, 351]). The odds of a specialist physiotherapist stratifying a subject as high risk were eight times higher than for a nonspecialist physiotherapist (OR 7.7; 95% CI [2.4, 24]).

## 4 DISCUSSION

This service evaluation reports the diagnostic and predictive value of physiotherapy assessment of suitability for extubation from Mechanical Ventilation (MV). It is unique in that the focus of the physiotherapists' assessment was to identify patients at high risk of extubation failure, and the aim was to determine accuracy of physiotherapists' prediction of extubation outcome. Previous studies have focused on early identification of patients suitable for extubation using protocolized care pathways and have not explored the clinical decision making of the therapists. In this cohort, physiotherapist classification as high risk and neurological inappropriateness were significantly associated with extubation failure, and specialist physiotherapists were able to predict extubation failure with high sensitivity.

### 4.1 Predictive ability of physiotherapists

Specialist physiotherapists' ability to differentiate extubation outcome was more accurate than that of nonspecialist physiotherapists. The sensitivity of specialist physiotherapists to predict extubation failure was 100% indicating that all patients who failed extubation had been deemed high risk of failure by the specialist physiotherapist. This is both financially and clinically significant as the risks associated with extubation failure include increased incidence of VAP, twofold increase in ICU‐LOS, reduced likelihood of hospital discharge to home, and increased mortality of up to 50% following reintubation (Frutos‐Vivar et al., 2011; Menon et al., 2012; Thille et al., 2011; Torres et al., 1995). Therefore, sensitivity to detect extubation failure should arguably be valued more highly than specificity in the clinical setting.

The logistic regression predictor model demonstrated the highest specificity to detect extubation failure (96%) but its overall accuracy was no better than that of the specialist physiotherapists (75% and 76%, respectively). In a clinical context, superior sensitivity is likely to be more desirable and the ability of an experienced clinician to determine where the balance of risk lies for an individual patient may be augmented by a mathematical risk predictor but is unlikely to be replaced.

The 100% sensitivity of specialist physiotherapists could be contrasted with the 33% sensitivity of physicians to predict extubation failure reported by Thille et al. (2015). In that study, a four‐item logistic regression model based on cough strength, secretion abundance, duration of intubation, and cardiac function significantly outperformed the physicians for accuracy of extubation failure prediction Area under the receiver operating curve (AUROC) 0.72 vs. 0.78, *p* = .04). Abundance of secretions and duration of intubation were associated with physiotherapist high risk stratification in this service evaluation, although neither were significantly associated with extubation outcome.

Nonspecialist physiotherapists had a much lower sensitivity to predict extubation failure; however, similar to the logistic regression model, their specificity was higher than the specialist physiotherapists. This could reflect that specialist physiotherapists were more cautious compared with their colleagues or they may have supported perceived high risk patients more effectively postextubation with fewer proportionately going on to experience extubation failure. A successfully managed high risk patient who went on to succeed extubation would be classed as a false positive in this evaluation. Nonspecialist physiotherapists were less likely to classify their patients as high risk that may reflect less familiarity with risk factors, reluctance to challenge the ICU consultant regarding the decision to extubate, or poorer clinical acumen compared with specialist physiotherapists.

Univariate logistic regression analysis indicated that physiotherapist stratification to high risk of extubation failure was more predictive of extubation failure than any other single predictor. Other than neurological inappropriateness, none of the assessed predictors were significantly associated with extubation failure in this cohort. As there were a significant number of patients who were extubated without a physiotherapy assessment, the full spectrum of predictive values may not have been collected. This may have contributed to the finding that single‐item predictors (such as P0.1, MIP, and PCEF) were not associated with extubation outcome in this cohort. An element of preselection may have occurred, with perhaps those patients deemed low risk being extubated prior to physiotherapy assessment and/or an assessment being specifically requested for those perceived higher risk of extubation failure.

### 4.2 Extubation failure rate

The extubation failure rate (29%) in this service evaluation was relatively high compared with the mean reported rate of 15% (Krinsley, Reddy, & Iqbal, 2012). This reported failure rate was based on early extubation failure. Taking this into consideration, the early extubation failure rate of 20% in this cohort is comparable. Similar to other reports, extubation failure compared with extubation success was associated with increased ICU‐LOS (Frutos‐Vivar et al., 2011) and ICU mortality (Menon et al., 2012). Absolute mortality was low at 7% due to the exclusion of tracheostomy and palliative extubations.

Had a high risk stratification by a physiotherapist been a barrier to extubation, 18 of the extubated patients would have remained intubated or been tracheotomized. In this scenario, the overall extubation failure rate would have been lower; however, it is worth noting that some patients would have remained intubated or undergone tracheostomy who could have been successfully extubated. Identifying a patient at high risk of extubation failure cannot be recommended as an absolute barrier to extubation but should be an indication for multidisciplinary care planning and risk–benefit assessment of options including supported extubation or tracheostomy.

### 4.3 Role of the physiotherapist

Physiotherapists are uniquely placed to optimize the patient's respiratory function both pre‐extubation by augmenting secretion clearance and postextubation by providing supportive interventions such as bridge NIV and airway clearance adjuncts (Gosselink et al., 2008). The results of this service evaluation demonstrate that specialist physiotherapists can detect patients who are at high risk of extubation failure with a high sensitivity following a thorough assessment. Although individual predictive indices were not associated with extubation outcome in this cohort, there is insufficient evidence to dismiss their utility. It is unclear how much the ability of the specialist physiotherapists to predict extubation outcome was influenced by these indices; however, as they are quick and inexpensive to perform at the bedside, they are recommended as part of a holistic assessment.

Having identified patients who are high risk of extubation failure, a multidisciplinary decision should be undertaken regarding the optimal management of such patients. This may include supported extubation with bridge NIV and airway clearance techniques such as mechanical in‐exsufflation or tracheostomy. Recent safety recommendations discourage tracheostomy without trial of extubation unless justification for tracheostomy is clearly documented (Wilkinson, Freeth, & Kelly, 2015). A thorough physiotherapy assessment as described by this service evaluation could provide objective evidence of such justification.

## 5 IMPLICATIONS FOR PHYSIOTHERAPY PRACTICE

Specialized physiotherapists can predict extubation failure with high sensitivity in the adult ICU. Stratification of patients as high risk of extubation failure by physiotherapists is significantly associated with extubation failure in this cohort. Physiotherapists are uniquely placed to support patients during the transition from mechanical support to liberation from the ventilator, and their expertise should be recognized through close collaboration with consultants regarding the timing of extubation.

### INSTITUTION

This study was performed at Guy's and St Thomas' NHS Foundation Trust, London, UK.

### PRIOR PRESENTATION

This study was presented by Gabriella Cork as an oral presentation of an abstract submission at the European Society of Intensive Care Medicine Annual Congress, September 2017, Vienna, Austria.

## CONFLICT OF INTEREST

The authors have no financial or otherwise conflicts of interest to declare.
